# Ablação Septal com Cateteres e Radiofrequência Guiada pela Ecocardiografia para Tratamento de Pacientes com Cardiomiopatia Hipertrófica Obstrutiva: Experiência Inicial

**DOI:** 10.36660/abc.20200732

**Published:** 2022-01-11

**Authors:** Bruno P. Valdigem, Edileide B. Correia, Dalmo A. R. Moreira, David Le Bihan, Ibraim Masciarelli Francisco Pinto, Alexandre A. Cunha Abizaid, Rogério Braga Andalaft, Antonio Tito Paladino, Halstead Alarcão Gomes Pereira da Silva, Joao Henrique Zucco Viesi

**Affiliations:** 1 Instituto Dante Pazzanese de Cardiologia São Paulo SP Brasil Instituto Dante Pazzanese de Cardiologia,São Paulo, SP – Brasil

**Keywords:** Cardiomiopatia hipertrófica obstrutiva, ablação septal, miectomia, ablação por cateter, obstrução de via de saída, gradiente

## Abstract

**Fundamentos:**

A cardiomiopatia hipertrófica (CMH) pode causar obstrução da via de saída do ventrículo esquerdo (VSVE) e ser responsável pelo surgimento de sintomas limitantes, como cansaço físico. Quando tais sintomas são refratários ao tratamento farmacológico, os tratamentos alternativos intervencionistas podem ser úteis, como a ablação septal por meio da infusão de álcool na artéria coronária ou por meio da miectomia cirúrgica. Recentemente, o uso de cateter de radiofrequência (RF) para ablação do septo endocárdico guiado por mapeamento eletroanatômico mostrou-se eficaz apesar da elevada incidência de bloqueio atrioventricular total. Uma alternativa seria a aplicação de radiofrequência no ponto de início do gradiente septal guiada pelo ecocardiograma transesofágico (ETE). O ecocardiograma é um método de imagem com elevada acurácia para determinação da anatomia septal.

**Objetivo:**

Avaliar o efeito em longo prazo da ablação septal para alívio do gradiente ventrículo-arterial, utilizando o ETE para auxiliar no posicionamento do cateter na área de maior obstrução septal. Avaliar também os efeitos da ablação na classe funcional e parâmetros ecocardiográficos.

**Métodos:**

Doze pacientes sintomáticos com obstrução da VSVE, refratários à terapia farmacológica, foram submetidos à ablação endocárdica septal com cateteres com ponta de 8 mm, cujo posicionamento foi orientado na região de maior obstrução com auxílio do ETE. Foram realizadas aplicações de radiofrequência (RF) termocontrolada e escalonadas sobre a área alvo. Após cada aplicação, o gradiente era reavaliado e nova aplicação era realizada de acordo com critério clínico. Foram avaliados os efeitos das aplicações de RF tanto para o gradiente em repouso como para o provocado por meio da manobra de Valsalva, e considerado o gradiente. As diferenças foram significativas quando o valor de p foi menor ou igual a 0,05.

**Resultados:**

Observou-se que a redução da média dos gradientes máximos obtidos foi de 96,8±34,7 mmHg para 62,7±25,4 mmHg ao final de três meses do procedimento (p=0,0036). Após um ano, a média dos gradientes máximos obtidos foi de 36,1±23,8 mmHg (p=0,0001). O procedimento foi bem tolerado e não houve registro de bloqueio atrioventricular total e nem complicações graves.

**Conclusão:**

A ablação septal guiada pelo ETE foi eficaz e segura, com resultados mantidos durante o período de seguimento clínico. É uma opção razoável para o tratamento intervencionista da obstrução da VSVE em CMH.

## Introdução

A cardiomiopatia hipertrófica obstrutiva (CMHO) é uma doença de origem genética que se manifesta por intensa hipertrofia miocárdica, além de fibrose de extensão variável. A obstrução da via de saída do ventrículo esquerdo (VSVE) é uma condição anatômica com um comportamento dinâmico e pode causar sintomas, como limitação aos esforços, além de ser responsável por casos de morte súbita. O tratamento intervencionista proposto para essa condição pode ser a alcoolização de ramos septais da artéria coronária ou a miectomia cirúrgica.^1^ Os resultados desses procedimentos ainda são variados na literatura e adotados de forma pouco frequente na prática clinica.^2,3^ Recentemente, a aplicação localizada de radiofrequência (RF) por meio de cateter, e empregando mapeamento eletroanatômico para melhor caracterização do septo interventricular, mostrou-se eficaz devido ao melhor controle da extensão da lesão e o mais preciso posicionamento do cateter na região mais espessada do septo interventricular. Essa abordagem parece ser mais segura, principalmente no que diz respeito ao comprometimento do sistema de condução que trafega pelo septo.^4-6^

### Ablação septal por cateter de radiofrequência

A miectomia cirúrgica traz consideráveis índices de morbidade e mortalidade, especialmente em cenários nos quais não há um centro de referência, com experiência, que inclua pelo menos mais de dez cirurgias ao ano. Isso motivou o desenvolvimento de alternativas menos invasivas, como a alcoolização septal.^7^

Apesar da menor morbidade pós-operatória e a necessidade de unidades de pós-operatório com menos estrutura, entusiastas da alcoolização também se depararam com limitações significativas. A necessidade de anatomia coronariana favorável (que pode não ser encontrada em até 20% dos candidatos à alcoolização) e a imprevisibilidade da extensão do dano miocárdico reduzem a aplicabilidade rotineira desta técnica.

Estudos recentes demonstram que a aplicação localizada de RF por meio de cateter tem se mostrado eficaz devido ao melhor controle da extensão da lesão e à localização mais precisa do cateter na região mais espessa do septo interventricular. Os cateteres utilizados foram os mesmos usados em eletrofisiologia invasiva para tratamento de arritmias ventriculares e atriais. Os investigadores utilizaram mapeamento eletroanatômico intracavitário para determinar a região septal a ser abordada, além do posicionamento do cateter, respectivamente, visando reduzir o risco de comprometimento do sistema de condução.^8-10^ A ablação foi realizada em sala de eletrofisiologia ou hemodinâmica, com acesso ao septo interventricular por via aórtica retrógrada ou via transeptal (e raramente com abordagem associada ao septo direito). Os cateteres utilizados em estudos prévios foram irrigados ou com ponta de 8mm em adultos. Os resultados foram variados, principalmente pelas técnicas utilizadas para abordagem do gradiente.^9-11^

Encontramos apenas um estudo de ablação por cateter de radiofrequência na população pediátrica, utilizando mapeamento eletroanatômico associado à ecocardiografia tranesofágica.^12^

A ablação por cateteres de radiofrequência permite facilidade de acesso da terapia a gradientes localizados em porções basais do ventrículo esquerdo, bem como a gradientes intraventriculares, portanto, não é limitada por anatomia coronariana. O uso do ecocardiograma transesofágico (ETE) permite a localização do ponto da obstrução em tempo real durante o procedimento.

### Hipótese

A ablação por cateter utilizando técnicas de eletrofisiologia com posicionamento do cateter terapêutico com ponta de 8 mm, com o auxílio do ETE, é um método simples e que permite a localização precisa da área septal crítica para estabelecer o gradiente ventrículo-aórtico. Essas premissas devem tornar essa técnica mais simples, eficaz e segura para a redução da obstrução da VSVE.

## Objetivos

### Objetivo primário

Avaliar a segurança e a eficácia da aplicação de RF no septo interventricular de pacientes com CMHO por meio de cateteres com ponta de 8 mm cujo posicionamento na área alvo fora guiado pelo ETE visando à redução de gradiente em VSVE.

### Objetivo secundário

Avaliar a repercussão da ablação septal ventricular sobre a sintomatologia, baseada na classe funcional, além de efeitos sobre o eletrocardiograma e ecocardiograma transtorácico antes e após três meses e um ano do procedimento.

## Materiais e métodos

Como prova de conceito, optamos por realizar um procedimento piloto em uma paciente sintomática de 63 anos de idade com contraindicação para abordagem por hemodinâmica e risco cirúrgico elevado. A paciente (não incluída nesta série) apresentava gradiente residual médio ventricular pós-miectomia cirúrgica. A mesma foi submetida à ablação por cateter com sucesso na área alvo, com redução de gradiente inicial de 100 mmHg para menos de 25 mmHg, resultado mantido após 24 meses de seguimento. Foi utilizada abordagem transeptal, em princípio, mas a via de acesso foi modificada para a via aórtica retrógrada pela instabilidade do cateter e pela dificuldade em acessar o local proposto para ablação. A ressonância magnética de controle três meses após a intervenção identificou a lesão de radiofrequência, provocada com a utilização de cateteres, adjacente à lesão causada durante o ato cirúrgico.^13^

### Seleção dos pacientes

Foram selecionados 12 pacientes com CMHO da Seção de Cardiomiopatias do Instituto Dante Pazzanese de Cardiologia, com sintomas refratários ao tratamento farmacológico. A seleção dos 12 pacientes se deu por adequação aos critérios de inclusão e exclusão e interesse em participação no projeto de pesquisa, bem como disponibilidade para participação nas consultas presenciais e exames subsequentes do protocolo. Todos foram submetidos aos seguintes exames: eletrocardiograma, ecodopplercardiograma transtorácico, além de perfil sanguíneo incluindo hemograma, glicemia de jejum, ureia, creatinina, coagulograma.

Os critérios de inclusão foram: indivíduos com cardiomiopatia hipertrófica obstrutiva sintomática, com gradiente provocado pela manobra de Valsalva maior ou igual a 50 mmHg a despeito de tratamento medicamentoso (ou pacientes com gradiente acima de 30 mmHg com necessidade de uso concomitante de vasodilatadores); contraindicação médica para miectomia (risco cirúrgico elevado em discussão da equipe de cardiologia ou por opção do paciente); ou contraindicação para alcoolização septal por parâmetros técnicos. Foram excluídos: portadores de marca-passo definitivo ou cardiodesfibriladores implantáveis, pois existe influência do modo de estimulação e dos parâmetros no gradiente ventricular. Também dificultaria a mensuração da avaliação dos efeitos da ablação no sistema de condução cardíaco, mascarando bloqueios atrioventriculares tardios. Além disso, foram excluídas pessoas com fibrilação atrial, já que a mensuração do gradiente se torna pouco reprodutível com a irregularidade da frequência cardíaca. Já que as medidas seriam necessárias para a decisão de interromper a ablação ou continuar aplicando, optou-se por remover esta variável na fase inicial do protocolo. Foram também excluídos pacientes com infecção em atividade com histórico de arritmia ventricular complexa, ou com histórico de morte súbita recuperada pela provável indicação de implante de cardiodesfibrilador ao longo do seguimento. O ecocardiograma transtorácico em repouso foi realizado pela manhã, com os pacientes em uso de suas medicações habituais.

A manobra de Valsalva foi realizada por examinador experiente, que explicou sua execução detalhadamente aos pacientes. Os registros ecocardiográficos foram obtidos durante toda a fase de esforço e relaxamento, e foram considerados os maiores valores de gradiente. A execução correta da manobra foi definida pelo avaliador, por meio da redução da pré-carga, definida pela redução da onda E do fluxo mitral em uma primeira tentativa. Somente quando o paciente estava executando adequadamente a manobra, em outro momento, era realizado o registro dos gradientes em via de saída. Não houve controle por meio de barômetros / fluxômetros.

O protocolo e os termos de consentimento foram aprovados pelo Comitê de Ética em Pesquisa e estão disponíveis na Plataforma Brasil (CAAE: 72754617.0.0000.5462; número do protocolo – CEP 4769/2017)

### Procedimento ablativo

Os procedimentos foram realizados em sala dedicada de eletrofisiologia, por eletrofisiologistas e cardiologistas com área de atuação em ecocardiografia, e com supervisão de equipe de anestesiologia.

As estratégias utilizadas por outros pesquisadores basearam-se na premissa de que o ponto mais espesso do septo era o mesmo onde se iniciava o gradiente. O gradiente, por sua vez, é composto pelo movimento sistólico anterior do folheto anterior da válvula mitral, movimento do músculo papilar e septo. Esses dados anatômicos, ponto no qual se inicia o gradiente, podem ser observados com maior confiabilidade pelo ecocardiograma durante a ablação.

Todos os procedimentos foram realizados sob anestesia geral. Foram realizadas uma punção em artéria femoral direita com introdutor 8F para acesso à região septal do ventrículo esquerdo via aórtica retrógrada; duas punções em veia femoral direita com introdutor 6F para posicionamento de cateter quadripolar em septo direito; e identificação do eletrograma de feixe de His, além de outro cateter quadripolar em ponta de ventrículo direito. Em seguida mensurou-se o gradiente ventrículo-aórtico, assim como foi localizado o ponto de maior aceleração do fluxo com o ecocardiograma transesofágico, caracterizando a área de maior obstrução. O ecocardiograma transesofágico foi realizado sob anestesia geral, com imagens obtidas inicialmente em posição esofágica. Quando necessário, com avaliação de posição em eixo curto, a sonda foi movimentada para a posição transgástrica. O exame foi realizado de acordo com o protocolo da American Society of Echocardiography,^14^ observando-se as cavidades em múltiplas angulações (0, 30^o^, 45^o^, 60^o^, 90^o^) para avaliação anatômica. A imagem ecocardiográfica permitiu a identificação da ponta do cateter terapêutico, que foi justaposta pelo eletrofisiologista à região septal, onde foi identificado o turbilhonamento de fluxo (*Aliasing*). O ecocardiograma, então, foi utilizado para observar a distância entre o folheto anterior da válvula mitral e a estabilidade do contato com a região de interesse.

**Figura 1:**
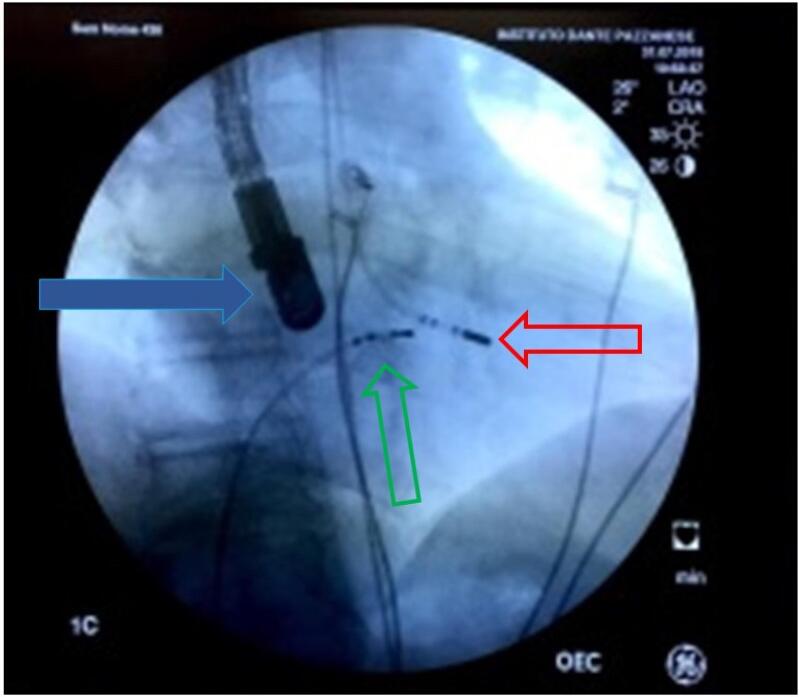
**–** Fluorsocopia em projeção obliqua anterior direita a 30 o, na qual se observa a sonda do ETE(seta azul, alem dos cateteres na região do feixe de His(seta verde) e o cateter de RF(terapêutico) em vermelho.

Com auxílio da fluoroscopia e registro de potenciais elétricos intracavitários com polígrafo TEB (Tecnologia Eletrônica Brasileira, São Paulo, Brasil) ou EPtracer (Cardiotek, Holanda), o cateter quadripolar deflectível foi posicionado em região de feixe de His; da mesma forma, um cateter quadripolar foi colocado na região apical do ventrículo direito, e um cateter terapêutico bidirecional 8F com ponta de 8mm foi posicionado em ventrículo esquerdo por via retroaórtica. O cateter terapêutico foi impactado na região septal de VE (conforme descrito anteriormente) no ponto de maior aceleração do fluxo sanguíneo, local no qual foi aplicada radiofrequência por 120 segundos (80W, 60^o^C), seguida de nova aferição do gradiente por meio de cateter de hemodinâmica, além do auxílio do ecocardiograma. A cada aplicação que resultasse na redução de pelo menos 25% do gradiente inicial foram adicionadas quatro novas aplicações em regiões adjacentes, observando-se a distância de pelo menos 1 cm do eletrograma de feixe de His (Figura 1).

Quando o gradiente máximo atingiu redução de pelo menos 25% do valor inicial, o procedimento foi interrompido e novas medidas foram realizadas em 10 e 20 minutos. Ao final do exame, reavaliou-se o gradiente intraventricular e o refluxo mitral, além de descartar complicações pós=procedimento.

Após retirada de introdutores, os pacientes permaneceram em unidade de terapia intensiva (UTI) por 24 horas, sendo encaminhados para enfermaria por 3 a 5 dias, com avaliação de troponina nas primeiras 48 horas. Realizou-se uma avaliação ecocardiográfica com 24 horas e no sexto dia de pós-operatório (para identificação de derrame pericárdico ou trombos pós-procedimento). Após seis semanas, três meses, seis meses e 12 meses, o paciente foi reavaliado clinicamente. O ecocardiograma transtorácico foi repetido no terceiro e no 12^o^ mês.

### Análise estatística

A distribuição das variáveis contínuas foi avaliada pelo teste de normalidade de Shapiro-Wilk, para análise comparativa dos tempos (pré-ablação, com três, meses e pré-ablação, com 12 meses). Para as variáveis com distribuição normal utilizou-se o teste T de Student pareado, e os resultados foram apresentados como média e desvio-padrão. Para aquelas com distribuição não normal utilizou-se o teste de Wilcoxon pareado, e os resultados foram apresentados como mediana e intervalo interquartil. Assim, somente a análise da espessura do septo ao final de 12 meses foi realizada com o teste de Wilcoxon. As demais variáveis foram analisadas utilizando o Teste de Mann-Whitney. Em todas as conclusões obtidas por meio das análises inferenciais o nível de significância considerado foi α = 5%. A classe funcional foi a única variável categórica analisada, e não foi utilizado teste estatístico para comparação pré e pós-procedimento, visto que todos os indivíduos apresentaram melhora. O software utilizado para as análises estatísticas foi o R^r^ (Viena, Áustria).

## Resultados

**Figura 2:**
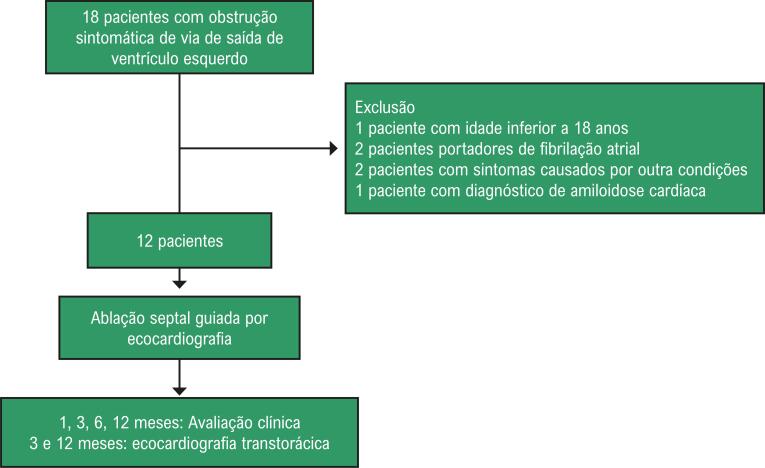
– Fluxograma de seleção e acompanhamento dos pacientes.

Dezoito pacientes foram pré-selecionados. Um deles foi excluído por ter menos de 18 anos; dois, por relato de fibrilação atrial paroxística ou presente no momento do procedimento; outros dois por sintomas possivelmente relacionados a outras causas que não o gradiente; e um indivíduo foi excluído com diagnóstico de amiloidose (Figura 2).

Os 12 pacientes incluídos correspondiam a nove mulheres e três homens. As características clínicas e ecocardiográficas dessa população são apresentadas na Tabela 1. Clinicamente, os sintomas dos pacientes foram relacionados ao elevado gradiente.

**Tabela 1:** – Características clínicas e ecocardiográficas da população avaliada

Idade	57,3 ± 3 anos
Altura	1,65 m ± 3,3
Peso	87 kg ± 15
Sexo Feminino	75%
HAS	75%
Diabetes mellitus	8%
Gradiente inicial em repouso mmHg	73,6 ± 38,1
Gradiente inicial provocado mmHg	96,8 ± 23,8
Classe funcional III ou IV	100%
FEVE média	67,0 ± 4
Espessura septal (mm)	21 **±** 6,4
Átrio esquerdo(ml)	65,4**±** 29,7
Uso de betabloqueadores	100%
Bloqueadores de canais de cálcio	33%
FC na admissão hospitalar	59,88 ± 4,19 bpm

*HAS: hipertensão arterial sistêmica; FEVE: fração de ejeção do ventrículo esquerdo; FC: frequência cardíaca.*

### Ablação

**Figura 3:**
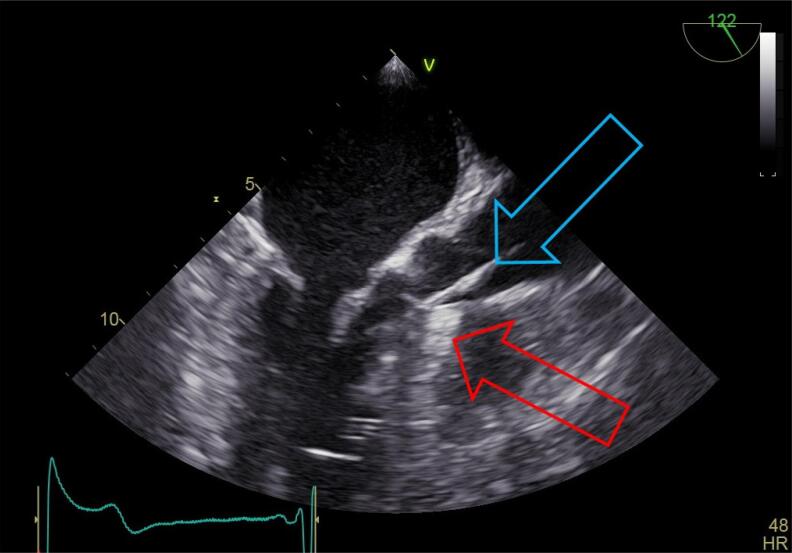
– *Ecocardiografia transesofágica perioperatória, na qual podem ser observados o septo interventricular refringente (seta vermelha) e o cateter terapêutico de radiofrequência (RF) que, após ultrapassar a válvula aórtica, é impactado no septo (seta azul). Esta região é definida pelo ecocardiografista como sendo a de maior gradiente. Nesse ponto, a RF é aplicada e, se reduzido o gradiente em pelo menos 25%, novas aplicações são repetidas ao redor da primeira aplicação, estendendo-se a área de lesão.*

A média da duração dos procedimentos foi de três horas. O posicionamento do cateter terapêutico indicado pelo ecocardiografista correspondia à região de maior gradiente, conforme demonstrado na Figura 3.

A média dos gradientes máximos iniciais mensurada durante o procedimento foi de 89 mmHg (±25,45). Ao seu término, a média reduziu para 36,9 mmHg (± 15,29). Surgiram três casos de bloqueio de ramo esquerdo relacionados ao procedimento sem incremento do intervalo HV (todos os pacientes permaneceram com intervalo HV menor ou igual a 60 ms). Não houve bloqueio atrioventricular transitório ou prolongamento do intervalo PR em nenhum dos casos. Não houve derrame pericárdico nem eventos embólicos cerebrais clínicos. Um paciente apresentou fístula arteriovenosa na região inguinal direita, que teve que ser corrigida cirurgicamente. Durante a internação, houve elevação de dosagem sérica de troponina em todos os pacientes. O valor máximo foi alcançado dentro das primeiras 12 horas e foi, em média, de 7,15 ng/dL (±4,36). O tempo médio de internação hospitalar foi de 5,8 dias (±2,7), por protocolo. Uma paciente ficou hospitalizada por 13 dias para avaliação de hematoma em região inguinal esquerda, que posteriormente foi diagnosticado e tratado como fístula arteriovenosa (Tabela 2).

**Tabela 2:** – Características individuais dos pacientes

paciente	idade	sexo	Medicações	Medicações	Gradiente Máximo	Gradiente Máximo	Gradiente Máximo	Classe funcional	Classe funcional	Complicações

			pré	12m	pré	3m	12m	pré	12m	
1	60	F	Atenolol 100mg/dia	Atenolol 100mg dia, verapamil 80mg/dia	126	61	47	III	II	Fistula arteriovenosa femoral. Cirurgia vascular. BRE novo
2	51	F	Propranolol 240mg/dia	Propranolol 240mg/dia	83	75	32	III	I	
3	71	M	Atenolol 100mg/dia Disopiramida 250mg/dia	Propranolol 120mg/dia	100	53	99	III	II	
4	62	F	Propranolol 160mg/dia	Propranolol 100mg/dia	141	99	41	III	II	
5	58	F	Metoprolol 100mg/dia verapamil 160mg/dia	Metoprolol 150mg/dia	152	55	25	IV	II	BRE novo
6	51	M	Atenolol 100mg/dia diltiazem 180mg/dia	Atenolol 50mg/dia	88	95	17	III	I	
7	23	F	Propranolol 240mg/dia não tolerados por hipotensão	Sem medicações	41	28	24	III	I	
8	73	F	Propranolol 240mg/dia verapamil 360mg/dia	Propranolol 240mg/dia verapamil 360mg/dia	135	89	56	III	I	
9	79	F	Ditiazem 120mg/dia atenolol 360mg/dia	Atenolol 100mg/dia Ditiazem 120mg/dia	70	70	18	IV	I	BRE novo
10	64	F	Atenolol 100mg/dia	Atenolol 100mg/dia	87	ND	17	III	I	
11	55	M	Metroprolol 75mg/dia	Metroprolol 50mg/dia	59	30	40	III	I	
12	41	F	Atenolol 200mg/dia	Atenolol 50mg/dia	80	35	17	III	I	

*BRE: bloqueio de ramo esquerdo ND: não disponível.*

Tabela 2 - Características clínicas e ecocardiográficas dos pacientes antes e após a ablação. O gradiente descrito foi o máximo obtido mesmo após provocação.

### Acompanhamento

**Figura 4:**
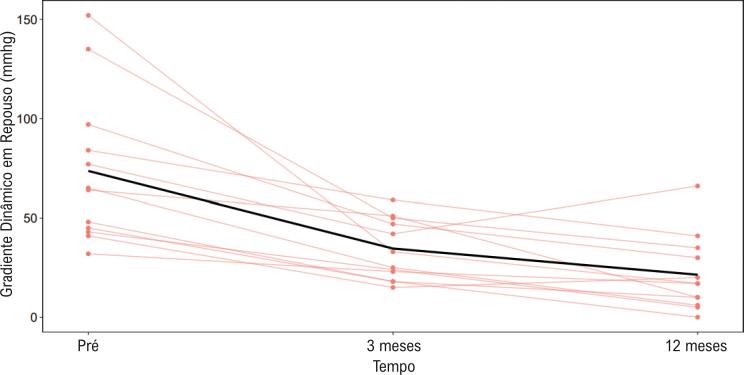
**–** Variação dos gradientes máximos em repouso pré-procedimento, após três meses e após 12 meses, para cada paciente. Observe a redução significativa do gradiente após a ablação, resultado que se manteve até os 12 meses de observação.

Houve redução dos gradientes provocados e em repouso em todos os pacientes persistentes, durante todo o acompanhamento. Os dados relativos à redução dos gradientes provocados e em repouso são apresentados nas Figuras 4 e 5. Por protocolo institucional, os pacientes continuaram recebendo a dose máxima de medicação tolerada até a obtenção de frequências cardíacas iguais ou abaixo de 60 bpm (sugerindo betabloqueio eficaz).

**Figura 5:**
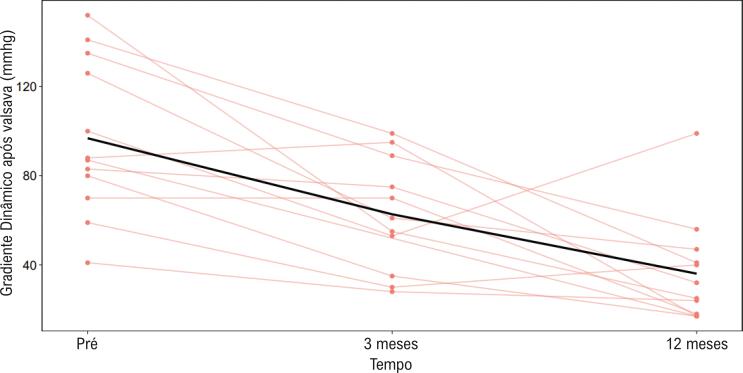
**–** Variação dos gradientes máximos pré-procedimento provocados pela manobra de Valsalva após três meses e após 12 meses, para cada paciente. Do mesmo modo, como observado no gradiente repouso, houve redução significativa do gradiente após ablação, resultado que se manteve até os 12 meses de observação.

Observou-se que a redução da média dos gradientes máximos obtidos foi de 96,8±34,7 mmHg para 62,7±25,4 mmHg ao final de três meses do procedimento (p=0,0036). Após um ano, a média dos gradientes máximos obtidos foi de 36,1±23,8 mmHg (p=0,0001).

**Figura 6:**
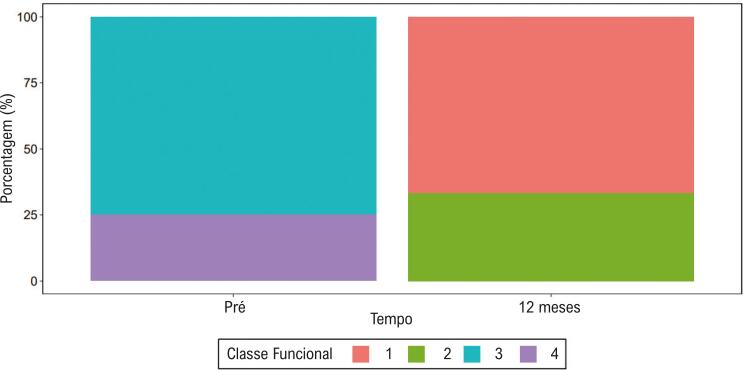
**–** Variação de classe funcional pré e ao término do seguimento de um ano.

Ao todo, 75% dos pacientes declaravam classe funcional de NYHA III, e 25% reportavam classe funcional IV, previamente ao procedimento de ablação. Ao final de um ano de seguimento após o procedimento, 66,7% estavam em classe funcional I, e 33,3% em classe funcional II (Figura 6).

Em relação a alterações eletrocardiográficas, não houve mudanças que justificassem alterar o tratamento, como bloqueios atrioventriculares ou supradesnivelamento de segmento ST. Os três pacientes que apresentaram alteração tiveram bloqueio de ramo esquerdo durante o procedimento, que persistiu ao longo do seguimento de um ano (identificados na Tabela 3). Exceto por diâmetro diastólico final do VE ao final de três meses, mas não de um ano do seguimento (que consideramos um evento isolado), não houve alteração significativa dos demais parâmetros ecocardiográficos avaliados nos três tempos (pré-operatório, ao final de três meses e ao final de um ano). A média dos parâmetros e a significância estatística estão disponíveis na Tabela 3.

**Tabela 3:** – Características e evolução dos parâmetros ecocardiográficos

	Antes do procedimento	3 meses	p (3 meses)	12 meses	p 12 meses
Raiz da aorta	32,2 +/- 2,5	31,9 +/- 2,5	0,6618	32,4 +/- 2,7	0,3251
Átrio esquerdo (mm)	47,2 +/- 6,8	47,9 +/- 8,6	0,8100	46,8 +/- 6,5	0,3803
Volume indexado do AE (ml)	65,4 +/- 29,7	60,1 +/- 15,1	0,0777	53,1 +/- 12,8	0,1144
Volume Diastólico Final do VE (mm)	42,4 +/- 4,3	44,1 +/- 3,4	0,0390	45,1 +/- 4,1	0,0692
Diâmetro Sistólico do VE (mm)	25,9 +/- 3,6	27,3 +/- 2,9	0,1946	27,3 +/- 2,7	0,8182
Septo VE (mm)	21 +/- 6,4	20,9 +/- 4,5	0.4332	17,8 +/- 3,4	0,3017^a^
Massa VE (g)	372,2 +/- 136,3	384,3 +/- 119,3	0,2644	358,6 +/- 86,9	0,8208
FEVE (SIMPSON - %)	68,7 +/- 5,4	68,1 +/- 4,1	0,4053	69,6 +/- 5,6	0,9642

*DDVE: diâmetro diastólico do ventrículo esquerdo; DSVE: diâmetro sistólico do ventrículo esquerdo; FEVE: fração de ejeção do ventrículo esquerdo; VE: ventrículo esquerdo. ^a^ = Teste de Wilcoxon pareado. Os demais valores de p são do Teste T de Student pareado.*

## Discussão

Esse estudo mostra que a ablação septal ventricular guiada pelo ETE é factível, com resultados favoráveis na redução significativa do gradiente ventricular e com baixas taxas de complicações. Todos os pacientes avaliados se beneficiaram do procedimento, conforme demonstrado pela melhora da classe funcional.

A miectomia cirúrgica é um procedimento com meio século de vida,^7^ e ainda é a intervenção mais amplamente aceita para o alívio da obstrução da VSVE. Ainda que necessária, uma alternativa menos invasiva surgiu mas não foi amplamente adotada pelos cardiologistas: a ablação septal com etanol, que é uma técnica realizada em laboratório de cateterismo e pode ser uma alternativa razoável à miectomia. Os resultados de ambas as técnicas para a redução de gradiente ainda são controversos em relação à mudança no prognóstico.

A obstrução da ejeção do ventrículo esquerdo é quantificada em mmHg, frequentemente por meio da ecocardiografia transtorácica. A diferença de pressões observada antes e após o ponto de obstrução é chamada de gradiente, e o mesmo ocorre em até um terço dos pacientes com de cardiomiopatia hipertrófica.^1^ O gradiente máximo obtido por meio do Doppler pulsado e contínuo na via de saída de ventrículo esquerdo (em repouso e após a manobra de Valsalva) é utilizado como marcador de risco de morte súbita em calculadoras de prognóstico, como o modelo proposto em 2014 por Elliot et al. nas diretrizes da European Society of Cardiology.^15^ O modelo foi validado e posteriormente apresentado em congresso da mesma sociedade em 2017.^16^

A forma de avaliação ecocardiográfica mais utilizada é a mensuração do gradiente durante a realização da manobra de Valsalva. Alternativas menos utilizadas são compressão abdominal pelo médico ecocardiografista, avaliação durante teste de esforço e provocação farmacológica.^17,18^ O uso de manobra provocativa permite estimar com reprodutibilidade correta os gradientes máximos obtidos no esforço físico, assim como minimizar o impacto da variação diária do gradiente em situações como desidratação e sedação.^19^

O tratamento farmacológico com betabloqueadores e bloqueadores de canais de cálcio é a terapia inicial para controle de sintomas em pessoas com obstrução sintomática. Os últimos geralmente são reservados para pacientes refratários aos betabloqueadores, ou em associação, quando a frequência cardíaca (FC) alvo não foi atingida. Recomenda-se cautela com associação de medicamentos bradicardizantes em pacientes com cardiomiopatia hipertrófica, especialmente naqueles com gradientes de repouso acima de 80 mmHg e sinais de insuficiência cardíaca.^20^ Assim, dois terços dos nossos pacientes não utilizaram a associação dessas classes farmacológicas, por terem atingido a FC alvo e pelo risco inerente ao uso dos mesmos. Conforme o disposto, a população amostral deste trabalho recebeu associação medicamentosa apenas quando sua FC alvo estava acima de 60 bpm e quando houve tolerabilidade clínica ao tratamento associado, o que ocorreu em cerca de um terço da casuística. Assim, não houve redução significativa das doses de medicações utilizadas após o término de um ano de seguimento.

O uso de ablação por RF foi relatado em 2011,^4^ em um estudo que abriu caminho para poucos outros desde então. A morbidade e a mortalidade cirúrgica elevadas posicionam o procedimento realizado por RF guiada pelo ETE em um patamar de intervenção minimamente invasiva. Além disso, essa técnica permite o seu emprego em pacientes mais jovens (<35anos) restrição relacionada à alcoolização septal. O procedimento por RF também independe da posição de ramos de coronárias septais, e torna a extensão da lesão após a ablação previsível. Por outro lado, estima-se que a taxa de reintervenção após alcoolização septal seja próxima de 12% pela persistência de gradiente residual sintomático.^19^ Os resultados obtidos com a ablação por cateter de RF na nossa amostra se assemelham aos de estudos anteriores, que utilizaram a mesma técnica quanto à redução do gradiente VSVE. A redução variou de 59% a 85% em alguns deles.^4,5,8,9^

O protocolo de ablação em nosso estudo foi intencionalmente minimalista. Utilizamos a ablação apenas do lado esquerdo, por meio da abordagem retrógrada aórtica, guiada pela ETE. Dessa forma, seria facilmente reprodutível em muitos laboratórios. Optamos por guiar a ablação utilizando ecocardiografia com navegação por fluoroscopia, porque o gradiente máximo não se deve apenas ao septo mais espesso. Outras estruturas participam da obstrução, como o movimento anterior da válvula mitral e os músculos papilares. O ETE também foi útil para identificar as cordoalhas da valva mitral, evitando-se sua lesão e prevenindo a insuficiência dessa válvula.

Os estudos iniciais que empregaram a RF para redução do gradiente em CMHO utilizaram cateteres irrigados. Este tipo de cateter necessita de infusão contínua de soro fisiológico impulsionado por bomba de infusão específica, com o fluxo aproximado de 1.000 ml/h durante a aplicação. O fluxo de infusão é reduzido durante o mapeamento (momento em que o cateter está dentro do coração, mas não é liberada energia de radiofrequência), mas ainda neste momento o paciente recebe 120 ml/h. Em um artigo de revisão, três pacientes apresentaram edema pulmonar ou congestão após o procedimento.^8^ Os cateteres terapêuticos com ponta de 8 mm utilizados no nosso estudo não requerem irrigação para seu adequado funcionamento. Além disso, mesmo não sendo a nossa intenção, o uso de cateter não irrigado pode melhorar a relação custo-benefício quando comparado ao irrigado, e pode ser uma opção adequada para laboratórios que não possuem mapeamento eletroanatômico prontamente disponível.

A redução da classe funcional da NYHA foi o principal benefício decorrente da redução do gradiente ventrículo-arterial. Ao término de um ano, todos os pacientes que referiam sintomas compatíveis com classe funcional III ou IV de NYHA relatavam melhora dos sintomas, e estes foram caracterizados com classe funcional I ou II. Isso permitiu que os pacientes melhorassem seu desempenho nas atividades nas atividades de rotina, resultado semelhante aos publicados anteriormente com outros protocolos. A melhora da classe funcional durante o período de observação deste estudo (até o final do primeiro ano) sugere uma modificação permanente do estado funcional do septo ventricular esquerdo. Como a redução da espessura septal não foi significativa, acreditamos que a cicatrização do septo endocárdico esquerdo possa inibir o seu abaulamento em direção à VSVE. Acreditamos que a cicatrização do septo submetido à lesão por RF tenha inibido o abaulamento sistólico em direção à VSVE, reduzindo, assim, o gradiente. Esse mecanismo seria diferente do dano endocárdico transmural, que ocorre quando a ablação septal alcoólica^10^ ou a ablação percutânea por RF septal intramiocárdica por agulha é realizada.^11^

Quanto à segurança da técnica aqui utilizada, não houve nenhum caso de bloqueio atrioventricular. O intervalo HV não se prolongou-se acima dos 60 ms, mesmo nos três pacientes que apresentaram dano ao ramo esquerdo do feixe de His. Por outro lado, com a ablação endocárdica por RF guiada pelo mapeamento eletroanatômico, não houve relato de bloqueio atrioventricular em uma série de casos^8^e de até 21% dos casos na série de Lawentz et al.^4^ Em um estudo, o suporte do marca-passo agudo foi necessário em 17% dos pacientes após o procedimento, mas o número real pode estar subestimado, uma vez que o protocolo do estudo exigiu implante de cardioversor desfibrilador implantável (CDI) em todos os pacientes devido à extensa ablação em ambos os lados do septo.^5^ Acreditamos que o protocolo menos agressivo, com um limiar mais baixo de interrupção da aplicação da energia de RF sobre o septo (redução de 25% do gradiente inicial), associado a uma ablação guiada pelo ETE, pode ter desempenhado um papel importante na questão da segurança. Um estudo em crianças com o diagnóstico utilizou ecocardiografia perioperatória para localização do ponto onde havia maior abaulamento do septo para aplicação de RF, utilizando cateteres terapêuticos de ponta irrigada de 4 mm associados ao mapeamento eletroanatomico,^12^ enquanto outro utilizou tecnologia de integração de imagem de ecocardiografia intracardíaca associada ao mapeamento eletroanatômico (CARTOSOUND, Biosense Webstser, CA, EUA).^21^ Pela heterogeneidade de técnicas, não é possível comparar as complicações dos procedimentos guiados por ETE, guiados apenas por mapeamento eletroanatômico ou por uma combinação de ambos. A meta-análise de Poon et al. não foi capaz de estabelecer a correlação entre o uso da ecocardiografia e o sucesso ou complicações. No entanto, sugeriu um potencial benefício do uso de ecocardiografia intracardíaca associada ao mapeamento eletroanatômico na localização do alvo da ablação.^21^ Essa observação foi baseada na experiência obtida no estudo de Cooper et al., que também foi coautor da meta-análise.^21^Ainda que se observe gradiente residual máximo provocado superior a 50 mmHg em dois casos da série, consideramos que o protocolo inicial deve ser aprimorado, buscando melhores preditores de sucesso que possam ser observados ainda na cirurgia. O pequeno número de casos desta série inicial também teve como objetivo melhorar os sintomas e preservar a integridade da condução elétrica, com ablação precisa. Talvez, em estudos futuros, uma ablação mais extensa possa conferir a mesma segurança com redução maior dos gradientes em longo prazo. Ainda assim, o critério para reintervenção somente seria atingido em um dos pacientes (gradientes superiores a 50 mmHg sintomáticos apesar de tratamento clínico).

Ainda não existem estudos comparando alcoolização septal com ablação por RF. Alguns registros sugerem que a incidência de bloqueio atrioventricular total seja estimada em 10-15% dos pacientes submetidos à alcoolização septal, particularmente em pacientes com bloqueio de ramo esquerdo previamente ao procedimento. Além disso, algum grau de bloqueio atrioventricular transitório foi observado em cerca de 50% dos pacientes durante ou na primeira semana após o procedimento.^16,17^ A alcoolização septal também esteve relacionada à maior área de fibrose se comparada à miectomia cirúrgica e elevada probabilidade de arritmias ventriculares no pós-operatório^19^

Uma meta-análise recente que incluiu 74 pacientes de seis estudos relatou dois tamponamentos que necessitaram de tratamento.^22^ Não houve complicações maiores no nosso grupo. Um paciente apresentou fístula arteriovenosa que necessitou de correção cirúrgica nas primeiras duas semanas após o procedimento.

### Limitações

Embora os resultados sejam encorajadores, este é um estudo observacional com tempo de seguimento relativamente curto. Acreditamos que uma comparação de métodos (ablação septal por etanol, RF endocárdica ou miectomia) seria ideal, mas o número de pacientes submetidos a ambos os procedimentos deveria ser maior, idealmente randomizado e, por consequência, envolver múltiplos centros de pesquisa. Uma opção seria iniciar um estudo prospectivo randomizado, comparando-se um grupo intervenção contra outro sem intervenção (*sham study*), uma vez que o alívio dos sintomas é o objetivo final do tratamento. Um efeito placebo não pode ser afastado em um estudo de série de casos como este. Pelo número limitado de casos, não foi possível quantificar a probabilidade de surgimento de arritmias ventriculares complexas em seguimento mais longo (ainda que no período de um ano nenhum dos pacientes tenha apresentado justificativa para implante de cardiodesfibrilador ou marca-passo definitivo).

## Conclusão

A ablação endocárdica por RF guiado pelo ETE é um procedimento eficaz, seguro em longo prazo, que reduz o gradiente da VSVE e melhora o grau funcional em pacientes com obstrução grave.
