# Supplementary material for: Ablação Septal com Cateteres e Radiofrequência Guiada pela Ecocardiografia para Tratamento de Pacientes com Cardiomiopatia Hipertrófica Obstrutiva: Experiência Inicial
Source: Arq Bras Cardiol. 2022 Jan 11;118(5):861–72. [Article in Portuguese] doi: 10.36660/abc.20200732 (PMC9368870; doi:10.36660/abc.20200732)
Supplement: Translation

# Translation: Septal Ablation with Radiofrequency Catheters Guided by Echocardiography for Treatment of Patients with Obstructive Hypertrophic Cardiomyopathy: Initial Experience

**Authors:** Bruno P. Valdigem, Edileide B. Correia, Dalmo A. R. Moreira, David Le Bihan, Ibraim Masciarelli Francisco Pinto, Alexandre A. Cunha Abizaid, Rogério Braga Andalaft, Antonio Tito Paladino, Halstead Alarcão Gomes Pereira da Silva, Joao Henrique Zucco Viesi

**Affiliations:** 1 Brazil Instituto Dante Pazzanese de Cardiologia,São Paulo, SP – Brazil

**Keywords:** Hypertrophic cardiomyopathy, LVOT obstruction, RF ablation, Myectomy, alcohol septal ablation

## Abstract

**Background:**

Hypertrophic cardiomyopathy (HCM) can cause obstruction in the left ventricular outflow tract (LVOT), and be responsible for the onset of limiting symptoms, such as tiredness. When such symptoms are refractory to pharmacological treatment, interventionist alternative therapies can be useful, such as septal ablation through the infusion of alcohol in the coronary artery or through myectomy. Recently, the use of a radiofrequency (RF) catheter for endocardial septal ablation guided by electroanatomic mapping has proven to be efficient, despite the high incidence of complete atrioventricular block. An alternative would be the application of RF at the beginning point of the septal gradient guided by the transesophageal echocardiography (TEE). The echocardiography is an imaging method with high accuracy to determine septal anatomy.

**Objective:**

To assess the long term effect of septal ablation for the relief of ventricular-arterial gradient, using TEE to help place the catheter in the area of larger septal obstruction. Besides, to assess the effects of ablation on the functional class and echocardiographic parameters.

**Methods:**

Twelve asymptomatic patients, with LVOT obstruction, refractory to pharmacological therapy, underwent endocardial septal ablation with 8mm-tip catheters, whose placement was oriented in the region of larger obstruction, assisted by the TEE. Temperature-controlled and staggered RF applications were performed. After each application, the gradient was reassessed and a new application was performed according to the clinical criterion. The effects of RF applications were assessed both for the gradient at rest and for that provoked by the Valsalva maneuver, and considering the gradient. The differences were significant when p-value was lower than or equal to 0.05.

**Results:**

It was possible to observe that the mean reduction of the maximum gradients was from 96.8±34.7 mmHg to 62.7±25.4 mmHg three months after the procedure (p=0.0036). After one year, the mean of maximum gradient was 36.1±23.8 mmHg (p=0.0001). The procedure was well tolerated, without records of complete atrioventricular block nor severe complications.

**Conclusion:**

The TEE-guided septal ablation was efficient and safe, and the results were maintained during the clinical follow-up period. It is a reasonable option for the interventionist treatment of LVOT obstruction in HCM.

## Introduction

Obstructive hypertrophic cardiomyopathy (OHCM) is a genetic condition that manifests itself through myocardial hypertrophy, besides fibrosis of variable extension. The obstruction of the left ventricular outflow tract (LVOT) is an anatomical condition with dynamic behavior, and can cause symptoms such as limitation to efforts, besides being responsible for cases of sudden death. The interventionist treatment proposed for this condition can be alcohol injection in the septal branches of the coronary artery or myectomy.^1^ The results of these procedures are still varied in the literature, and little adopted in the clinical practice.^2,3^ Recently, the RF applied locally through a catheter, and using the electroanatomic mapping for a better characterization of the interventricular septum, has proven to be efficient due to the better control of the lesion extension and the more accurate placement of the catheter in the thicker region of the interventricular septum. This approach seems to be safer, especially regarding the impact on the conduction system of the septum.^4-6^

### Septal ablation by radiofrequency catheter

Myectomy has considerable morbidity and mortality rates, especially in scenarios in which there is no reference, experienced center that includes at least more than ten surgeries a year. This motivated the development of less invasive alternatives, such as septal alcoholization.^7^

Despite the lower postoperative morbidity and the need for postoperative units with less structure, advocates of alcoholization are also faced with significant limitations. The need for a favorable coronary anatomy (which cannot be found in up to 20% of the candidates to alcoholization) and the unpredictability of the extension of myocardial damage reduce the chances of applying this technique as a routine.

Recent studies show that the RF applied locally through a catheter has been efficient due to the better control of the injury extension and to the more precise placement of the catheter in the thicker region of the interventricular septum. These catheters were the same ones used in invasive electrophysiology for the treatment of ventricular and atrial arrhythmia. The researchers used intracavitary electroanatomic mapping to establish the septal region to be approached, besides the position of the catheter, respectively, aiming at reducing the risk of affecting the conduction system.^8-10^ The ablation was performed in an electrophysiology or hemodynamic room, with access to the interventricular septum through retrograde aortic or transseptal path (rarely with an approach associated with the right septum). The catheters used in previous studies were irrigated, or had an 8mm-tip in adults. The results ranged, especially because of the techniques used to approach the gradient.^9-11^

We found only one study about RF catheter ablation in the pediatric population, using the electroanatomic mapping associated with transesophageal echocardiography.^12^

The RF catheter ablation allows the easy access of therapy to gradients located in the basal portions of the left ventricle, as well as to intraventricular gradients; therefore, it is not limited by coronary anatomy. The use of a transesophageal echocardiography (TEE) allows the real time location of the point of obstruction in real time during the procedure.

### Hypothesis

Catheter ablation using electrophysiology techniques, by placing the therapeutic 8mm-tip catheter with the help of the TEE, is a simple method that allows the precise localization of the critical septal area to establish the ventricular-aortic gradient. These premises should make this technique simpler, more efficient and safer for the reduction of the LVOT obstruction.

## Objectives

### Primary Objective

To assess the safety and efficacy of RF application in the interventricular septum of patients with OHCM through 8mm-tip catheters whose placement on the target area was guided by TEE, aiming at reducing the gradient in LVOF.

### Secondary Objective

To evaluate the repercussion of ventricular septal ablation on symptomatology, based on the functional class, besides the effects on electrocardiography and on the transthoracic echocardiogram before and three months and one year after the procedure.

### Materials and methods

As proof of concept, we chose to conduct a pilot procedure in a symptomatic 63-year old patient, who had contraindication for an approach by hemodynamics and high surgical risk. The patient (not included in this series) presented mean post-myectomy residual ventricular gradient. The individual underwent a successful catheter ablation in the target area, with reduction of the initial gradient from 100 mmHg to less than 25 mmHg, and this result was maintained after 24 months of follow-up. The transseptal approach was used at first, but the access path was changed to the retrograde aortic path due to the instability of the catheter and the difficulty to access the proposed site for ablation. A magnetic resonance for control three months after the intervention identified the RF injury, caused by the use of catheters, adjacent to the lesion caused during the surgery.^13^

### Patient selection

Twelve patients with OHCM were selected from the Cardiomyopathy Section of Instituto Dante Pazzanese de Cardiologia, with refractory symptoms to the pharmacological treatment. The 12 patients were selected for being in accordance with the inclusion and exclusion criteria, and for being interested in participating in the study, as well as being available to attend appointments and subsequent examinations of the protocol. They were all submitted to the following examinations: electrocardiography (EKG), transthoracic echocardiogram with doppler, besides blood profile including blood count, fasting glycemia, urea, creatinine, coagulogram.

The inclusion criteria were: individuals with symptomatic OHCM, whose gradient is provoked by a Valsalva maneuver higher than or equal to 50 mmHG, despite the treatment (or patients whose gradient is higher than 30 mmHg, with the need for the concomitant use of vasodilators); medical contraindication for myectomy (high surgical risk established by the cardiology staff or by patient’s choice); or contraindication for septal alcoholization due to technical parameters. The excluded participants were: those with a definitive pacemaker or implantable cardioverter defibrillator, since they influence the mode of stimulation and the parameters in the ventricular gradient. It also would make it difficult to measure the effects of ablation in the heart conduction system, masking late atrioventricular blocks. Besides, we excluded individuals with atrial fibrillation, since the measurement of the gradient becomes little reproducible due to the irregularity of the heart rate (HR). Since the measurements would be necessary for the decision of interrupting ablation or continuing to apply it, the choice was to remove this variable in the initial stage of the protocol. Patients with ongoing infections and history of complex ventricular arrhythmia, or with a history of sudden death recovered due to the probable indication of an implantable cardioverter defibrillator during follow-up, were also excluded. The transthoracic echocardiography at rest was performed in the morning, and patients were on their usual medications.

The Valsalva maneuver was performed by an experienced examiner, who explained its execution to the patients in detail. The echocardiographic records were obtained throughout the effort and relaxation stages, and were considered as the highest gradient values. The proper execution of the maneuver was defined by the evaluator, through the pre-load reduction, defined by the reduction of the mitral flow E-wave in the first attempt. Only when the patient was executing the maneuver properly, at another time, the gradients were recorded in the outflow. There was no control using barometers/flow meters.

The protocol and consent forms were approved by the Research Ethics Committee and are available in Plataforma Brasil (CAAE: 72754617.0.0000.5462; protocol number – CEP 4769/2017).

### Ablation

The procedures were performed in a dedicated electrophysiology room, by electrophysiologists and cardiologists working with echocardiography, and with the supervision of the anesthesiology staff.

The strategies used by other researchers were based on the premise that the thicker point of the septum was the same where the gradient began. The gradient, in turn, is composed of the systolic anterior movement of the anterior leaflet of the mitral valve, movement of the papillary movement and septum. These anatomic data, point in which the gradient begins, can be observed with more accuracy by the echocardiogram during the ablation.

All of the procedures were performed under general anesthesia. A right femoral artery puncture was performed with an 8F guiding catheter to access the septal region of the left ventricle via retrograde aorta; two right femoral vein punctures with a 6F guiding catheter were performed to place the quadripolar catheter in the right septum; and we identified the His axis electrogram, besides another quadripolar catheter tip in the right ventricle. Then we measured the ventricular-aortic gradient and located the point of highest acceleration of the flow with the transesophageal echocardiography, characterizing the area of larger obstruction. The transesophageal echocardiography was performed under general anesthesia, and the images were initially obtained in the esophageal position. When necessary, with the evaluation of the short axis position, the probe was moved to the transgastric position. The examination was performed according to the protocol of the American Society of Echocardiography,^14^by observing the cavities in several angles (0, 30^o^, 45^o^, 60^o^, 90^o^) for the anatomical evaluation. The echocardiographic image allowed to identify the tip of the therapeutic catheter, which was overlapped to the septal region by the electrophysiologist, when it was possible to identify the aliasing. The echocardiography was then used to observe the distance between the anterior leaflet of the mitral valve and the stability of the contact with the region of interest.

With the help of fluoroscopy and the record of intracavitary electrical potentials with a TEB (Tecnologia Eletrônica Brasileira, São Paulo, Brazil) or EPtracer (Cardiotek, Netherlands) polygraph, the deflectable quadripolar catheter was placed in the His axis region; likewise, a quadripolar catheter was placed on the apical region of the right ventricle, and a bidirectional 8F therapeutic catheter with an 8mm-tip was placed in the left ventricle through the retroaortic course. The therapeutic catheter was impacted on the septal region of the LF (as previously described), in the point of the highest acceleration of the blood flow, where the RF was applied for 120 seconds (80W, 60^o^C), followed by a new measurement of the gradient through a hemodynamic catheter, besides the assistance of the echocardiogram. To each application that resulted in the reduction of at least 25% of the initial gradient, four new applications were added to adjacent regions, observing the distance of at least 1 cm of the His axis electrogram (Figure 1).

**Figure 1:** – *Fluoroscopy in a 30º right anterior oblique position, in which it is possible to observe the TEE probe (blue arrow), besides the catheters in the His axis region (green arrow) and the RF catheter (therapeutic). para Fluoroscopy in a 30º right anterior oblique position, in which it is possible to observe the TEE probe (blue arrow), besides the catheters in the His axis region (green arrow) and the RF catheter (therapeutic) - red arrow*

When the maximum gradient reached a reduction of at least 25% of the initial value, the procedure was interrupted and new measures were taken in 10 and 20 minutes. At the end of the examination, the intraventricular gradient and the mitral reflux were reassessed, besides ruling out post-procedure complications.

After removing the introducers, the patients remained in the intensive care unit (ICU) for 24 hours, and were referred to the nursery for 3 to 5 days, with an assessment of troponin in the first 48 hours. Then, an echocardiography was performed 24 hours and six days after the procedure (to identify pericardial effusion or post-procedure thrombosis). After six weeks, three months, six months and 12 months, the patients were clinically reassessed. The transthoracic echocardiogram was repeated on the third and on the 12nd month.

### Statistical analysis

The distribution of the continuous variables was assessed by the Shapiro-Wilk normality test for the comparative analyses of periods (pre-ablation, after three months, and pre-ablation, after 12 months). For the variables with normal distribution, the paired Student’s t-test was used and the results were presented as mean and standard deviation. For those with non-normal distribution, the paired Wilcoxon test was used, and the results were presented as median and interquartile range. Thus, only the septal thickness analysis after 12 months was carried out with the Wilcoxon test. The other variables were analyzed using the Mann-Whitney test. In all of the conclusions obtained through the inferential analysis, the considered significance level was α = 5%. The functional class was the only analyzed categorical variable, and we did not use a statistical test for the pre and post-procedure comparison, considering that all individuals improved. The software used for statistical analyses was the R^r^ (Viena, Austria).

## Results

Eighteen patients were pre-selected. One of them was excluded for being less than 18 year of age; two for reporting paroxysmal atrial fibrillation or at the time of the procedure; two others, for having symptoms that were possibly related to other causes other than the gradient; and one individual was excluded due to a diagnosis of amyloidosis (Figure 2).

**Figure 2:** **–** Flowchart showing patient selection and follow-up.

The 12 included patients corresponded to nine women and three men. The clinical and echocardiographic characteristics of this population are presented in Table 1. Clinically, the patients’ symptoms were related to a high gradient.

**Table 1:** – Clinical and echocardiographic characteristics of the assessed population

Age	57.3 ± 3 years
Height	1.65 m ± 3.3
Weight	87 kg ± 15
Female gender	75%
SAH	75%
Diabetes mellitus	8%
Initial gradient at rest - mmHg	73.6 ± 38.1
Initial provoked gradient - mmHg	96.8 ± 23.8
Functional class III or IV	100%
Mean LVEF	67.0 ± 4
Septal thickness (mm)	21 **±** 6.4
Left atrium (ml)	65.4**±** 29.7
Use of betablockers	100%
Calcium channel blockers	33%
HR at hospital admission	59.88 ± 4.19 bpm

*SAH: systemic arterial hypertension; LVEF: left ventricular ejection fraction; HR: heart rate.*

### Ablation

The mean duration of the procedures was three hours. The placement of the therapeutic catheter indicated by the echocardiography professional corresponded to the region with the highest gradient, as demonstrated in Figure 3.

**Figure 3:** – *Perioperative transesophageal echocardiography, which shows the refringent interventricular septum (red arrow) and the radiofrequency (RF) therapeutic catheter, which, after surpassing the aortic valve, is impacted on the septum (blue arrow). This region is defined by the echocardiography professional as the one with the highest gradient. At this point, RF is applied and, if the gradient is reduced in at least 25%, new applications are repeated around the first application, thus extending the lesion area.*

The mean of initial maximum gradients measured during the procedure was 89 mmHg (±25,45). At the end, this mean decreased to 36.9 mmHg (±15.29). There were three cases of left bundle branch block related to the procedure, without the increment of the HV interval (all patients presented HV interval lower than or equal to 60 ms). There were no transient atrioventricular block nor prolongation of the PR interval in any of the cases. Likewise, there were no pericardial effusion nor clinical embolic brain events. One patient presented with arteriovenous fistula in the right inguinal region, which had to be surgically corrected. During hospitalization, all patients received an increased serum dose of troponin. The maximum value was reached in the first 12 hours, and was, in average, 7.15 ng/dL (±4.36). The mean time of hospitalization was 5.8 days (±2.7), per protocol. One patient was hospitalized for 13 days for the assessment of a hematoma in the left inguinal region, which was then diagnosed and treated as arteriovenous fistula (Table 2).

**Table 2:** – Individual characteristics of the patients

Patient	Age	Sex	Medications	Medications	Maximum gradient	Maximum gradient	Maximum gradient	Functional class	Functional class	Complications

			pré	12m	pré	3m	12m	pré	12m	
1	60	F	Atenolol 100mg/day	Atenolol 100mg dia, verapamil 80mg/day	126	61	47	III	II	Femoral arteriovenous fistula. Vascular surgery. New LBBB
2	51	F	Propranolol 240mg/day	Propranolol 240mg/day	83	75	32	III	I	
3	71	M	Atenolol 100mg/day Disopiramida 250mg/day	Propranolol 120mg/day	100	53	99	III	II	
4	62	F	Propranolol 160mg/day	Propranolol 100mg/day	141	99	41	III	II	
5	58	F	Metoprolol 100mg/day verapamil 160mg/day	Metoprolol 150mg/day	152	55	25	IV	II	New LBBB
6	51	M	Atenolol 100mg/day diltiazem 180mg/day	Atenolol 50mg/day	88	95	17	III	I	
7	23	F	Propranolol 240mg/day not tolerated by hypotension	No medications	41	28	24	III	I	
8	73	F	Propranolol 240mg/day verapamil 360mg/day	Propranolol 240mg/day verapamil 360mg/day	135	89	56	III	I	
9	79	F	Ditiazem 120mg/day atenolol 360mg/day	Atenolol 100mg/day Ditiazem 120mg/day	70	70	18	IV	I	New LBBB
10	64	F	Atenolol 100mg/day	Atenolol 100mg/day	87	ND	17	III	I	
11	55	M	Metroprolol 75mg/day	Metroprolol 50mg/day	59	30	40	III	I	
12	41	F	Atenolol 200mg/day	Atenolol 50mg/day	80	35	17	III	I	

*LBBB: left bundle branch block. NA: not available.*

Table 2. Clinical and echocardiographic characteristics of patients before and after ablation. The described gradient was the maximum obtained even after being provoked.

### Follow-up

There was a reduction of the provoked and at rest gradients in all persistent patients during follow-up. The data regarding the reduction of provoked and at rest gradients are presented in Figures 4 and 5. According to institutional protocol, the patients continued to receive the maximum dose of tolerated medication until their HRs were equal to or lower than 60 bpm (suggesting an efficient betablock).

**Figure 4:** **–** Variation of maximum gradients at rest and pre-procedure, after three and 12 months, for each patient. It is possible to observe the significant reduction of the gradient after ablation, and this result was maintained until the 12 months of observation.

**Figure 5:** – *Variation of maximum pre-procedure gradients provoked by the Valsalva maneuver after three and 12 months, for each patient. Likewise, as observed in the at rest gradient, there was significant reduction of the gradient after ablation, and this result was maintained until the 12 months of observation.*

It was observed that the reduction of the mean of maximum gradients was from 96.8±34.7 mmHg to 62.7±25.4 mmHg three months after the procedure (p=0.0036). Após um ano, a média dos gradientes máximos obtidos foi de 36,1±23,8 mmHg (p=0,0001).

Overall, 75% of the patients declared being in NYHA functional classification III, and 25% reported functional classification IV before the ablation procedure. One year after the procedure, 66.7% were in functional classification I, and 33.3%, II (Figure 6).

**Figure 6:** **–** Variation of functional classification before and after the one-year follow-up.

Regarding electrocardiographic changes, there were none that could justify a different treatment, such as atrioventricular blocks or ST segment depression. The three patients that presented with changes had left bundle branch block during the procedure, which persisted throughout the one-year follow-up period (identified in Table 3). Except for the final diastolic diameter of the LV at the end of three months, but not one year of follow-up (which we consider as an isolated event), there was no significant change in the other echocardiographic parameters assessed in the three periods (preoperative, after three months and after one year). The mean of the parameters and the statistical significance are available in Table 3.

**Table 3:** – Echocardiographic characteristics before the procedure, after three and 12 months. Mean and standard deviation

	Before the procedure	3 months	p (3 months)	12 months	p 12 months
Aortic root	32.2 +/- 2.5	31.9 +/- 2.5	0.6618	32.4 +/- 2.7	0.3251
Left atrium (mm)	47.2 +/- 6.8	47.9 +/- 8.6	0.8100	46.8 +/- 6.5	0.3803
Indexed volume of the LA (ml)	65.4 +/- 29.7	60.1 +/- 15.1	0.0777	53.1 +/- 12.8	0.1144
Final diastolic diameter of the LV (mm)	42.4 +/- 4.3	44.1 +/- 3.4	0.0390	45.1 +/- 4.1	0.0692
Final systolic diameter of the LV (mm)	25.9 +/- 3.6	27.3 +/- 2.9	0.1946	27.3 +/- 2.7	0.8182
LV septum (mm)	21 +/- 6.4	20.9 +/- 4.5	0.4332	17.8 +/- 3.4	0.3017^a^
LV mass(g)	372.2 +/- 136.3	384.3 +/- 119.3	0.2644	358.6 +/- 86.9	0.8208
LVEF (SIMPSON - %)	68.7 +/- 5.4	68.1 +/- 4.1	0.4053	69.6 +/- 5.6	0.9642

*LVDD: left ventricle diastolic diameter; LVSD: left ventricle systolic diameter; LVEF: left ventricular ejection fraction; LV: left ventricle. ^a^: paired Wilcoxon test. The other p-values are from the paired Student’s t-test.*

## Discussion

This study shows that TEE-guided ventricular septal ablation is feasible, with favorable results in the significant reduction of the ventricular gradient, with low complication rates. All of the assessed patients benefitted from the procedure, as demonstrated by the improved functional classification.

Myectomy has been a reality for fifty years,^7^ and it is still the most widely accepted intervention to relieve the obstruction of the LVOF. It is necessary, but a less invasive alternative has appeared, but has not been widely adopted by cardiologists: septal ablation with ethanol, which is a technique performed in the catheterization laboratory and can be a reasonable alternative to myectomy. The results of both techniques to reduce the gradient are still controversial regarding the change in prognosis.

The obstruction of the left ventricular ejection is quantified in mmHg, often through the transthoracic echocardiography. The difference of pressures observed before and after the point of obstruction is called a gradient, and the same occurs in up to one third of the patients with hypertrophic cardiomyopathy.^1^ The maximum gradient obtained through the pulse and the continuous Doppler in the LVOF (at rest and after the Valsalva maneuver) is used as a marker for risk of sudden death in calculators of prognosis, such as the model proposed in 2014 by Elliot et al. in the guidelines of the European Society of Cardiology.^15^ The model was validated and then presented at a congress of the same society, in 2017.^16^

The most used form of echocardiographic evaluation is the measurement of the gradient during the Valsalva maneuver. Less used alternatives are abdominal compression by the echocardiography physician, evaluation during an exercise stress test and pharmacological induction.^17,18^ The use of a provocative maneuver allows to estimate the proper reproducibility of maximum gradients obtained at physical effort, as well as to minimize the impact of the daily variation of the gradient in situations such as dehydration and sedation.^19^

The pharmacological treatment with betablockers and calcium channel blockers is the initial therapy to control symptoms in people with symptomatic obstruction. The later are usually indicated for patients who are refractory to betablockers, or in association, when the target HR is not reached. It is important to be careful about the association of bradycardizing drugs in patients with hypertrophic cardiomyopathy, especially those whose gradients at rest are higher than 80 mmHg, with signs of heart failure.^20^ Therefore, two thirds of our patients did not use the association of these pharmacological classes, since they reached the target HR and due to the inherent risk of using them. As shown, the sample of this study was only submitted to drug association when the target HR was above 60 bpm, and when there was clinical tolerance towards the associated treatment, which occurred in about one third of the sample. Therefore, there was no significant reduction of medication doses used after the conclusion of the one-year follow-up period.

The use of RF ablation was reported in 2011,^4^ in a study that made way to a few others since then. The high morbidity and mortality surgical rates established the procedure of TEE-guided RF as a minimally invasive intervention. Besides, this technique can be used in younger patients (<35 years), and such a restriction is related to the septal alcoholization. The RF procedure is not dependent on the position of septal coronary branches, and makes the extension of the post-ablation lesion predictable. On the other hand, it is estimated that the rate of reintervention after septal alcoholization is close to 12% due to the persistence of the symptomatic residual gradient.^19^ The results obtained with the RF catheter ablation in our sample are similar to those of previous studies, which used the same technique as to the reduction of the LVOF gradient. The reduction ranged from 59% to 85% in some of them.^4,5,8,9^

The ablation protocol in our study was intentionally minimalist. We used ablation only on the left side, through the retrograde aortic approach, guided by TEE. Therefore, it would be easily reproducible in many laboratories. We chose to guide the ablation using echocardiography with fluoroscopy, because the maximum gradient is not only owed to the thicker septum. Other structures participate in the obstruction, such as the anterior movement of the mitral valve and papillary muscles. TEE was also useful to identify the mitral valve strand, thus preventing its lesion and failure.

The initial studies that used RF to reduce the gradient in OHCM used irrigated catheters. This type of catheter requires a continuous infusion of physiological serum pumped by a specific infusion pump, and approximate flow of 1,000 ml/h during the application. The infusion flow is reduced during mapping (moment when the catheter is inside the heart, but the radiofrequency energy is not released), but even in this moment the patient receives 120 ml/h. In a review article, three patients presented with pulmonary edema or congestion after the procedure.^8^ The therapeutic catheters with 8-mm tips used in our study do not require irrigation for functioning properly. Besides, even if it was not our intention, the use of a non-irrigated catheter can improve the cost-benefit relationship when compared to the irrigated one, so it can be a viable option for laboratories that do not have electroanatomic mapping promptly available.

The reduction of the NYHA functional classification was the main benefit resulting from the reduction of the ventricular-arterial gradient. After one year, all patients who referred having symptoms compatible with NYHA functional classifications III or IV reported improvement in the performance of activities of daily living, and similar results were found in other studies. The improvement of the functional classification during the observation period of this study (until the end of the first year) suggests a permanent modification of the functional status of the left ventricular septum. Since the reduction of the septal thickness was not significant, we believe that the healing of the left septal endocardium may inhibit its bulging towards the LVOF. We believe that the healing of the septum submitted to the RF lesion has inhibited the systolic bulging towards the LVOF, thus reducing the gradient. This mechanism would be different from the endocardial, transmural damage, which occurs when the alcoholic septal ablation^10^ or the needle percutaneous intramyocardial septal RF ablation are performed.^11^

As to the safety of the technique used here, there were no cases of atrioventricular block. The HV interval was not prolonged for more than 60 ms, even in the three patients that presented damage in the left branch of the His axis. On the other hand, with the endocardial ablation by RF guided by electroanatomic mapping, there were no reports of atrioventricular block in a case series^8^and in up to 21% of the cases in the series by Lawentz et al.^4^ In a study, the support of the acute pacemaker was necessary in 17% of the patients after the procedure, but the real number can be underestimated, once the study protocol required an implantable cardioverter defibrillator (ICD) in all patients due to the extensive ablation in both sides of the septum.^5^ We believe that the less aggressive protocol, with a lower threshold for interrupting the application of RF energy on the septum (reduction of 25% from the initial gradient), associated with TEE-guided ablation, may have played an important role in the matter of safety. A study with children with the diagnosis used perioperative echocardiography to locate the site with larger septal bulge to apply the RF, using therapeutic catheters with irrigated 4mm-tips associated with electroanatomic mapping,^12^ whereas another analysis used an intracardiac echocardiography image integration technology associated with electroanatomic mapping (CARTOSOUND, Biosense Webstser, CA, USA).^21^ Due to the heterogeneity of techniques, it is not possible to compare the complications of the TEE-guided procedures, those guided only by electroanatomic mapping, or by a combination of both. The meta-analysis by Poon et al. was not able to establish a correlation between the use of echocardiography and the rates of success or complications. However, it suggested a potential benefit of the use of intracardiac echocardiography associated with electroanatomic mapping in the localization of the target of the ablation.^21^ This observation was based on the experience obtained in the study by Cooper et al., who was also a co-author of the meta-analysis.^21^Even if we can observe a maximum residual provoked gradient higher than 50 mmHg in two cases of the series, we consider that the initial protocol should be improved, in search of better success predictors that can be observed still in surgery. The small number of cases in this initial series also aimed at improving the symptoms and preserving the integrity of the electric conduction, with precise ablation. Maybe, in future analyses, a more extensive ablation can provide the same safety and lead to a higher reduction of gradients in the long term. Still, the criterion for reintervention would only be reached in one of the patients (symptomatic gradients higher than 50 mmHg, despite clinical treatment).

There are no studies comparing septal alcoholization and RF ablation. Some records suggest that the incidence of complete atrioventricular block is estimated in 10-15% of the patients who undergo septal alcoholization, especially those with left bundle branch block before the procedure. Besides, some level of transient atrioventricular block was observed in about 50% of the patients during or one week after the procedure.^16,17^ Septal alcoholization was also related to the larger area of fibrosis if compared to myectomy, and high changes of ventricular arrhythmias in the postoperative period.^19^

A recent meta-analysis that included 74 patients of six studies reported two cases of tamponade that required treatment.^22^ There were no major complications in our group. One patient presented with arteriovenous fistula, which required surgical correction in the two weeks after the procedure.

### Limitations

Even though the results are encouraging, this is an observational study, whose follow-up time is relatively short. We believe that a comparison between methods (septal ablation with ethanol, endocardial RF or myectomy) would be ideal, but the number of patients submitted to both procedures should be higher, ideally randomized and, as a consequence, involving multiple research centers. An option would be to start a randomized prospective study and compare a group with intervention and another one without intervention (sham study), once the relief of symptoms is the final objective of the treatment. A placebo effect cannot be ruled out in a case series study such as this one. Due to the limited number of cases, it was not possible to quantify the probability of complex ventricular arrhythmias in the long term (even if none of the patients has presented a justification for an implantable cardioverter defibrillator or definitive pacemaker in a one-year period).

## Conclusion

TEE-guided RF endocardial ablation is an efficient procedure, safe in the long term, which reduces the gradient of LVOF and improves the functional level of patients with severe obstruction.
